# Clinical, Molecular and Serological Diagnosis of Canine Leishmaniosis: An Integrated Approach

**DOI:** 10.3390/vetsci7020043

**Published:** 2020-04-14

**Authors:** Maria Paola Maurelli, Antonio Bosco, Valentina Foglia Manzillo, Fabrizio Vitale, Daniela Giaquinto, Lavinia Ciuca, Giuseppe Molinaro, Giuseppe Cringoli, Gaetano Oliva, Laura Rinaldi, Manuela Gizzarelli

**Affiliations:** 1 Department of Veterinary Medicine and Animal Production, University of Naples Federico II, 80137 Naples, Italy; mariapaola.maurelli@unina.it (M.P.M.); boscoant@tiscali.it (A.B.); daniela.giaquinto@gmail.com (D.G.); lavinia_vet1@yahoo.com (L.C.); giuseppe.molinaro@unina.it (G.M.); giuseppe.cringoli@unina.it (G.C.); gaetano.oliva@unina.it (G.O.); laura.rinaldi@unina.it (L.R.); manuela.gizzarelli@unina.it (M.G.); 2 Regional Center for Monitoring Parasitic Diseases (CREMOPAR), Campania Region, 84025 Eboli (Sa), Italy; 3 National Reference Center for Leishmaniosis, Istituto Zooprofilattico Sperimentale della Sicilia, 90129 Palermo, Italy; fabrizio.vitale@izssicilia.it

**Keywords:** leishmaniosis, dog, loop-mediated isothermal amplification (LAMP), real-time polymerase chain reaction (Rt-PCR), immunofluorescence antibody test (IFAT), enzyme-linked immunosorbent assay (ELISA), SNAP *Leishmania* test

## Abstract

Canine leishmaniosis (CanL) is caused by protozoans of the genus *Leishmania* and characterized by a broad spectrum of clinical signs in dogs. Early diagnosis is of great importance in order to perform an appropriate therapy and to prevent progression towards severe disease. The aim of this study was to compare a point-of-care molecular technique, i.e., the loop-mediated isothermal amplification (LAMP), with a real-time polymerase chain reaction (Rt-PCR), and three serological techniques, i.e., immunofluorescence antibody test (IFAT), enzyme-linked immunosorbent assay (ELISA), and a rapid SNAP *Leishmania* test, to develop an integrated approach for the diagnosis of CanL. Sixty dogs were chosen after physical examination and collection of blood and sera samples, fine-needle aspiration of lymph nodes, and conjunctival swabs were performed. Lymphadenopathy (82.3%), as well as clinicopathological alterations of total proteins (70.6%), were the most frequent signs. Forty-one (68.3%) samples resulted positive at least to one technique. IFAT resulted in the best serological diagnostic method (specificity = 100%, sensitivity = 97.2%), detecting a higher number of positive samples than those revealed by other techniques. Among the samples used for molecular analysis, fine-needle aspiration of lymph nodes was revealed as the best sample source. LAMP showed a substantial agreement (*κ* = 0.80; *p* <0.0001) with Rt-PCR; therefore, it could be promising for the rapid diagnosis of CanL. Nevertheless, further studies should be performed to confirm these findings.

## 1. Introduction

Canine leishmaniosis (CanL) is an important vector-borne zoonotic parasitic disease caused by protozoans of the genus *Leishmania,* which are transmitted to dogs (and humans) by the bite of infected female phlebotomine sandflies [1]. The dog is considered the main reservoir of *Leishmania infantum* in endemic areas [2], and approximately 2.5 million dogs are affected by CanL in the Mediterranean and peri-Mediterranean areas each year [3,4].

CanL is characterized by a broad spectrum of clinical signs and degrees of severity, due to pathogenic mechanisms of *Leishmania,* and to the variable immune response of individuals. Hence, diagnosis is not easy and should be based on an integrated approach based on anamnesis, clinical signs, clinicopathological alterations, and usage of different laboratory techniques [5,6]. Moreover, early diagnosis of CanL is of great importance in order to perform an early and appropriate therapy and to prevent progression towards severe disease [7]. 

The main diagnostic methods for CanL are classified as parasitological, serological, and molecular tests [8]. Parasitological techniques consist of microscopic examination of different samples (bone marrow, lymph nodes, cutaneous lesions, etc.) and of highly specialized assays, e.g., parasite culture or xenodiagnosis, that usually are not used in the routine practice [9]. Serological techniques, including immunochromatographic test, immunofluorescent antibody test (IFAT), and enzyme-linked immunosorbent assay (ELISA), are the most common methods to detect exposed dogs [10]. In the last decade, molecular diagnostic assays became increasingly relevant and widespread. Molecular techniques include conventional PCR, nested-PCR, and quantitative real-time PCR (Rt-PCR) [11]. All the above-mentioned tools often are time-consuming, different in each lab, and the identification of parasites requires specialized personnel. Therefore, there is a need to develop a highly standardized, sensitive, specific, and rapid diagnostic method to reliably detect CanL.

The loop-mediated isothermal amplification (LAMP) is a promising technique developed by Notomi et al. [12] and characterized by an isothermal amplification for nucleic acid detection. LAMP has been applied to diagnose several pathogens, including bacteria [13,14], viruses [15,16], and different parasites, e.g., *Babesia* [17], *Plasmodium* [18], *Cryptosporidium* [19], *Trypanosoma* [20], *Giardia* [21], *Schistosoma japonicum* [22], and *Toxoplasma gondii* [23]. Moreover, several studies have successfully employed LAMP assay in the diagnosis of leishmaniosis in dogs, humans, and vectors [24,25,26], using different DNA targets, e.g., kinetoplast minicircle genes (kDNA), 18S ribosomal DNA (rDNA), ribosomal DNA internal transcribed spacer 1 (ITS1), K26 antigen-coding gene [27], and cysteine protease B (*cpb*) multicopy gene [28]. These authors showed that LAMP is easy-to-use, highly sensitive (90–98%) and specific (80–100%) and allows to perform in-situ analysis, but no standardized protocols are available in the veterinary field. Contrariwise, to detect human leishmaniosis, the Loopamp™ *Leishmania* kit has been developed by the Eiken Chemical Co. (Tokyo, Japan) and successfully validated [29,30,31,32]. 

Comparison studies between LAMP and serological techniques for CanL diagnosis have shown that LAMP has a higher specificity than ELISA and IFAT [24,28] and a higher sensitivity than ELISA [24], but lower than IFAT [28].

The aim of this study was to compare a commercial point-of-care LAMP kit, with an Rt-PCR protocol and three serological techniques (IFAT, ELISA, and a rapid SNAP *Leishmania* test) to develop an integrated approach for the diagnosis of CanL.

## 2. Materials and Methods

### 2.1. Study Area and Collection of Samples

This study was carried out with the approval of the University of Naples Federico II ethics committee (Protocol number: PG/2019/0133613). The Strengthening the Reporting of Observational Studies in Epidemiology (STROBE) checklist was used as a guideline for this study (https://www.strobe-statement.org/index.php?id=available-checklists) [33]. The study was conducted in the Campania region of southern Italy (Latitude = 39°59′15″–41°30′25″; Longitude = 13°45′25″–15°48′23″), a highly endemic CanL area, which extends over an area of 13,590 km^2^. The region is mainly hilly and extends from 0 to 1890 m above sea level. The climate is Mediterranean with dry summers and wet winters. The National Reference Center for Leishmaniosis (CReNaL) reported a seroprevalence of 20% of CanL in this region (unpublished data, 2018). From July 2018 to July 2019, 60 stray and owned dogs arrived for a physical individual examination. Special attention was paid to CanL signs, and a clinical form was filled out for each subject. The sampling was not performed using a statistical formula, but rather by choosing all the dogs (stray and owned) subjected to physical examination at the Veterinary Hospital of the University of Naples, excluding pregnant or lactating bitches, or subjects on immunosuppressive therapy, or treated for CanL in the previous six months. The owned dogs were submitted to physical examination after the signature of an informed owner’s consent. Of the 60 chosen dogs, 44 were stray dogs (73.3%; 95% CI = 60.1–83.6), whilst 16 were owned dogs (26.7%; 95% CI = 16.5–39.9). All the dogs were half-breed. 

From each dog, blood (in a tube with EDTA and a tube with serum separator gel), two conjunctival swabs, and fine-needle aspiration of lymph nodes were collected. All applicable international, national, and/or institutional guidelines for the care and use of animals were followed (i.e., Good Clinical Practice, VICHGL9, 2000; Directive 2010/63/UE; National Legislative Decree 26/2014). In the laboratory, each blood sample with EDTA was divided into three aliquots: the first for a complete blood count analysis (CBC), the second for LAMP, and the third for Rt-PCR analysis. Each blood sample in the serum separator tube was centrifuged at 360 g for 15 min and divided into two aliquots, one used for biochemical analysis (chemistry panel, protein electrophoresis) and one stored at −20 °C until serological analysis. 

Clinical information was not available to staff that performed molecular and serological analyses to ensure the unbiasedness of the results.

### 2.2. Molecular Analysis

DNA extractions from blood and lymph nodes were performed using the kit Leishmania Screen Glow (Avantech Group, Angri (Sa), Italy) following the producer’s instructions. An aliquot of each extracted DNA sample was stored at −20 °C until LAMP analysis, while a second aliquot was sent to the CReNaL to perform the Rt-PCR. For conjunctival swabs, DNA extraction was performed from one sample using the Kit Leishmania Screen Glow with a modification of the producer’s instruction. Briefly, a conjunctival swab was put in a 2 mL tube with 500 L of extraction buffer for 10 min at room temperature. The DNA, thus, obtained was amplified by LAMP. The second conjunctival swab was sent to the CReNaL for the Rt-PCR.

LAMP was performed using the above-mentioned kit, Leishmania Screen Glow, following the producer’s instructions. In each amplification run, one positive and one negative control (without DNA) was used (both supplied by the kit). Briefly, in each tube with lyophilized inner (FIB and BIP) and outer (F3 and B3) primers to amplify the 18SrRNA gene, plus a partial part of the internal transcribed spacer 1 (ITS-1), 22 μL of LAMP Mix, 30 μL of mineral oil and 3 μL of extracted DNA were added. Twelve reactions were incubated for each run for 60 min at 60 °C in an ICGene Vet detector, connected to a tablet with dedicated software to read the results. At the end of incubation, the amplifi cation of the target gene was confirmed, based on direct visual inspection of the graphic obtained; a positive amplification showed a sigmoid curve, while in the absence of amplification showed a straight line. The software used to visualize the amplification gave also an immediate indication of the result. For a positive sample, near the name of the sample appeared a ‘plus sign’, for negative a ‘minus sign’.

Rt-PCR was performed using primers to amplify a region of the minicircle kinetoplast DNA (kDNA) [34]. The serial dilutions consisted of equivalents of DNA from 1 × 10^6^ cells to 1 cell per amplified sample. Each amplification was performed in duplicate, in a 20 µL reaction mixture containing the Bio-Rad Universal Master Mix (Bio-Rad, Hercules, CA, USA), the specific primers, the probe in the optimized concentration and 2 μL (100 ng) of extracted DNA. The thermal cycling conditions included an initial incubation for 2 min at 50 °C for uracyl-N-glycosylase activity. This step was followed by a 10 min denaturation at 95 °C and 40 cycles of 95 °C for 15 s and 60 °C for 35 s. The reactions were performed in a LightCycler 96 Roche (Roche, Risch-Rotkreuz, Switzerland). To quantify parasite burdens, cycle threshold (Ct) values obtained for each test sample were compared with those obtained for the corresponding standard curve. A negative control (only PCR mix, without DNA) was added for each run to verify contaminations.

Data on molecular analysis were blinded for personnel that performed serological studies.

### 2.3. Serological Analysis

Serum samples were analyzed by IFAT, ELISA, and a rapid SNAP *Leishmania* test.

IFAT slides provided by CReNaL were prepared using promastigotes of strain MHOM/TN/80/IPT1. Anti-*Leishmania* antibodies were detected with anti-dog IgG conjugated to fluorescein isothiocyanate produced in rabbit (Sigma–Aldrich, St. Louis, MO, USA).

Samples were considered positive if they showed a titer ≥1:160. Reading was performed using a fluorescence microscope (Leica DM 2500, Wetzlar, Germany) by three independent persons. Positive and negative controls provided by CReNaL were added for each test to verify the validity of the results.

To perform ELISA, a commercial test was used (ID Screen Leishmaniasis Indirect, ID VET, Grables, France), following the producer’s instructions. Two replicates of negative and positive controls (both supplied by the kit) were added to each plate. The optical density (OD) of each sample was measured at 450 nm using a microplate reader (Thermo Fisher Scientific, Waltham, MA, USA). Following the producer’s instruction, the test was considered valid if the mean value of the optical density of positive controls was > 0.350, and the ratio between the mean value of positive controls and the mean value of negative controls was > 3. For each sample, the OD percentage was calculated; if it was ≤ 40% was considered negative; if it was 40% < OD% < 50% was doubt, whilst if it is ≥ 50% was positive.

Finally, a commercial rapid SNAP *Leishmania* test (IDEXX, Ludwigsburg, Germany) was used following the producer’s instructions. The sample was considered positive if the colored control and sample spots were visible.

The Standards for Reporting of Diagnostic Accuracy Studies (STARD) checklist (https://www.equator-network.org/reporting-guidelines/stard/) [35] was used to report results about the performance of the techniques.

### 2.4. Statistical Analysis

We considered true positive samples those that showed positivity with two or more techniques, while we considered true negative samples those resulted negative using all the techniques.

Sensitivity, specificity, negative and positive predictive values (NPV and PPV) were calculated for each molecular and serological technique, considering the combined results of each group of methods as a gold standard (molecular or serological). The gold standard was chosen on the proposition basis that when a reliable gold standard is not available, at least two different tests should be performed [6,36]. The agreement between LAMP and Rt-PCR was calculated using Cohen’s *κ* statistic [37]. The *κ* measure was interpreted as follows: 0, no agreement; 0.01–0.20, poor agreement; 0.21–0.40, fair agreement; 0.41–0.60, moderate agreement; 0.61–0.80, substantial agreement; and 0.81–1.0, nearly perfect agreement [37].

A chi-squared test was performed using the SPSS 23.0 software (IBM, Armonk, NY, USA) to study the association between positivity and dogs’ life conditions (stray or owned dogs). Moreover, a correlation analysis was carried out between the severity of symptoms or *Leishmania* amastigotes per mL levels and IFAT titers in positive dogs for CanL by bivariate correlations. The difference was considered significant at *p* < 0.05.

## 3. Results

### 3.1. Clinical Signs

Forty-seven out of the 60 dogs (78.3%; 95% CI = 65.5–87.5) showed overt clinical signs of CanL, whilst 13 (21.7%, 95% CI = 12.5–34.5) were asymptomatic. The clinical and clinicopathological alterations most frequently found in the 47 chosen dogs suspected to be positive for CanL are reported in Table 1.

Forty-one samples (68.3%, 95% CI = 54.9–79.4) resulted positive for at least one (serological or molecular) technique as described below, but only 35 (58.3%, 95% CI = 44.9–70.7) were true positive for CanL (one false positive was obtained with LAMP, three false positive were obtained with ELISA and two with SNAP *Leishmania* test). Of these positive dogs, five were asymptomatic dogs at physical examination (14.3%, 95% CI = 5.4–31.0); it should be noted that all these five dogs were positive with serological techniques and four of them also using molecular techniques. The positivity for CanL was not associated with live conditions (*p* > 0.05). 

### 3.2. Molecular Analysis 

Specificity, sensitivity, negative and positive predictive values (NPV and PPV), and time to obtain results, with LAMP and Rt-PCR, are reported in Table 2. Thirty-two samples resulted positive at least with one molecular technique and one sample type, but one of these resulted a false positive (one lymph node sample analyzed by LAMP). The highest number of positive samples was obtained using lymph node samples by both molecular techniques (29 true positive samples). Of these positive dogs, 21 (72.4%; 95% CI = 52.5–86.6) showed also lymphadenopathy at physical examination. Conjunctival swabs permitted to detect only 13 positive dogs and six (50.0%; 95% CI = 22.3–77.7) of them also showed ocular lesions at physical examination. Finally, three dogs resulted positive for CanL, only by Rt-PCR on blood samples.

**Figure 1:**
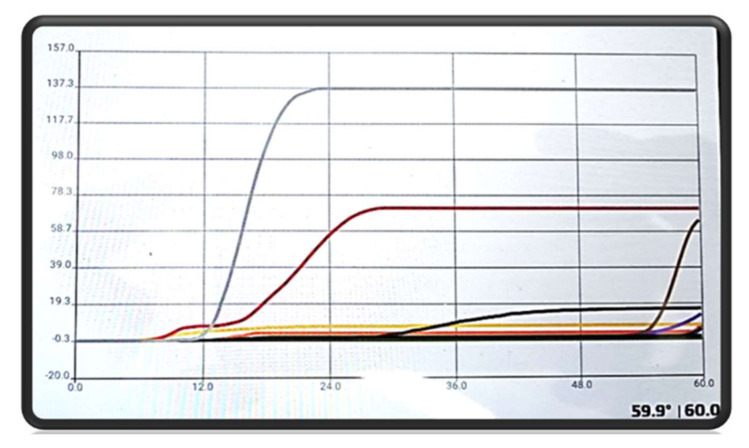
Results for positive samples, gray and red sigmoid curve, and negative samples. This graph was obtained using the software provided by Avantech Group and connected with the ICGene Vet detector. The time required is indicated on the x-axis is indicated, the fluorescence intensity is indicated on the y-axis.

When using LAMP, 26 lymph node samples (43.3%; 95% CI = 30.8–56.7), zero blood samples (0%; 95% CI = 0.2–7.5) and 12 conjunctival swabs (20.0%; 95% CI = 11.2–32.7) were positive for *Leishmania* (Figure 1). As reported in Table 3, two lymph node samples were positive by LAMP but negative by Rt-PCR, while of the corresponding sera, one was negative, and one was positive by serological techniques; therefore, one lymph node sample resulted in false positive by LAMP. Two conjunctival swabs were positive by LAMP, but negative by Rt-PCR and the corresponding sera were positive by serological techniques. 

When using Rt-PCR, 28 lymph node samples (46.7%; 95% CI = 33.9–59.9), three blood samples (5%; 95% CI = 1.3–14.8) and 11 conjunctival swabs (18.3%; 95% CI = 9.9–30.9) were positive for *Leishmania* with concentration ranged between 5 and 78,000 *Leishmania* amastigotes per mL (Leish/mL) (Table 4). As reported in Table 3, four lymph node samples were positive by Rt-PCR (with a concentration of 15–21,500 Leish/mL), but negative by LAMP, while the corresponding sera were positive by IFAT (titers 1:160–1:5120) and three of them also by ELISA and SNAP *Leishmania* test. Three blood samples were positive by Rt-PCR (10–150 Leish/mL), but negative by LAMP, while the corresponding sera were positive by ELISA and three of them also by SNAP *Leishmania* test. Finally, one conjunctival swab was positive by Rt-PCR (200 Leish/mL), but negative by LAMP and the corresponding serum was positive by IFAT (titer 1:1280), by ELISA and SNAP *Leishmania* test. 

LAMP and Rt-PCR showed a substantial *κ* agreement (*κ* = 0.80; *p* < 0.0001). 

### 3.3. Serological Analysis

**Figure 2:**
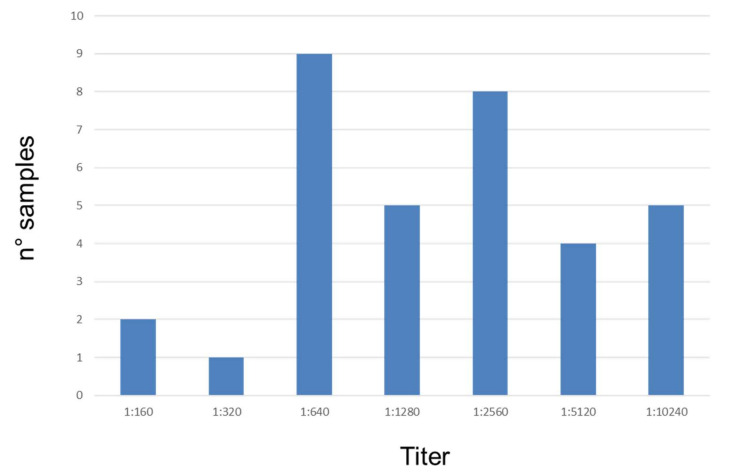
The number of dogs positive to *Leishmania* distributed by antibody titers.

Specificity, sensitivity, negative and positive predictive values and time to obtain the results for each serological technique are reported in Table 5. Thirty-four (56.7%; 95% CI = 43.3–69.2) sera resulted *Leishmania* positive by IFAT with titers ≥ 1:160 (Figure 2). No significant correlations were found between severity of symptoms (*r*= 0.2; *p* > 0.05) or number of amastigotes/mL (detected by Rt-PCR) (*r*= 0.2; *p* > 0.05) and IFAT titers in positive dogs for CanL. However, most dogs with IFAT titers ≥ 1:2560 showed a higher symptom score, whereas dogs with IFAT titers ≥1:1280 had a higher number of amastigotes/mL.

The ELISA test showed 35 (60.0%; 95% CI = 46.6–72.2) positive samples, plus one doubt sample. As reported in Table 6, three samples were false positive. In fact, these sera were negative by all the other techniques used (serological and molecular techniques), while one sample resulted in negative by ELISA and positive by IFAT with a titer of 1:160 and by Rt-PCR (lymph node matrix) with a concentration of 15 Leish/mL.

The SNAP *Leishmania* test showed 24 positive (40.0%; 95% CI = 27.8–53.5) samples. As reported in Table 6, two samples were false positive. In fact, these sera were negative by all other techniques, while 12 samples resulted in negative by the SNAP *Leishmania* test, but positive by IFAT (titers from 1:160 to 1:2560) and corresponding lymph nodes were positive by LAMP and by Rt-PCR (5–75,000 Leish/mL).

Only one dog resulted in positive by molecular techniques (Rt-PCR = 5 Leish/mL; LAMP = positive) but negative by serological techniques. Instead, four dogs resulted positive by serological techniques, but negative by molecular techniques.

## 4. Discussion

The diagnosis of CanL can represent a challenge for veterinarians because this parasitosis shows a wide spectrum of clinical forms from asymptomatic infection to a severe and life-threatening generalized disease, affecting any organ, tissue, or body fluid with different nonspecific clinical signs, referable to other diseases [5]. In this study, 47 (78.3%) of the 60 enrolled dogs showed clinical signs referable to CanL, 41 (68.3%) dogs resulted positive by at least to one diagnostic technique, but only 35 were true positive (58.3%) for CanL. It should be noted that five of these positive dogs were asymptomatic at physical examination. Therefore, the development of a reliable and rapid diagnostic technique, like the LAMP assay, could be important for the early management of infected dogs, but also to prevent zoonotic transmission of leishmaniosis to humans in endemic areas. The most frequent clinical and clinicopathological alterations found in these dogs were lymphadenopathy (82.3%), the increase of total protein values and low A/G ratio (70.6%), followed by skin lesions (58.8%). The abovementioned signs were all compatible with CanL, but not pathognomonic and could be present in other infections and/or coinfections. Thus, a potential limitation of the present study was the lack of serological detection of exposure to other pathogens that could present overlapping with leishmaniosis symptoms. However, the main aim of this study was to compare a commercial point-of-care LAMP kit with a Rt-PCR and three serological techniques: IFAT, a commercial ELISA, and a rapid SNAP *Leishmania* test to perform an integrated approach to diagnose CanL.

IFAT resulted as the best technique, detecting a higher number of true positive dogs (56.7%), as well as higher values of specificity (100%) and sensitivity (97.2%), while for ELISA a specificity of 88.0% and a sensitivity of 94.3% were found, in agreement with the OIE Manual on leishmaniosis [38]. However, the commercial ELISA used in our study is based on purified antigens of *L. infantum*, while recent papers showed that recombinant antigens (e.g., KMP11, LiP, rk39, rk26, rA2, rk9, rKE16, and histones such as H_2_A) improve the sensitivity and the specificity of the immunodiagnostic tests [39]. The number of positive dogs detected by the SNAP *Leishmania* test, as well as its sensitivity and specificity, were lower than the values found when using the other serological techniques (62.9% and 92.0% vs 100% and 97.2% for IFAT vs 94.3% and 88.0% for ELISA, respectively). 

As reported in Solano-Gallego et al. [5], rapid serological qualitative techniques provide only positive or negative results with a high risk of false negatives. Moreover, a positive sample with a qualitative technique needs to be also analyzed with a quantitative technique (i.e., IFAT) in clinical studies to understand the difference between pre and post-treatment. Nevertheless, IFAT evaluation is subjective, and its results depend on the experience of the personnel [6]. In addition, sensitivity and specificity of serological tests are very variable in the literature, also considering the choice of a gold standard. In fact, some authors considered the combination of at least two techniques [6,36], as in our study, while some other authors used a Bayesian method to evaluate the performances of the tests [10,40] 

In the last years, many molecular techniques have been developed for the diagnosis of leishmaniosis [25]. In particular, the Rt-PCR resulted very useful to evaluate follow-up, nevertheless, this molecular technique is not widely used due to its complexity, and it requires expensive laboratory equipment and expert staff [25,41].

Since 2009, different protocols of LAMP have been provided for the diagnosis of CanL in dogs. However, there is not a standardized protocol, so it could be very important to use a commercial standardized kit as in this study. LAMP resulted in a simple and rapid (1 h) technique that does not require expensive instruments and/or specialized staff. Moreover, a high tolerance towards contaminants and inhibitory components of this method was reported [42]. For these reasons, LAMP could be very useful for point-of-care diagnosis of CanL [43]. In our paper, lymph node samples resulted in the best sample source for LAMP, as well as for Rt-PCR. This is in agreement with Pennisi et al. [41] and Castagnaro et al. [44] that showed a lower sensitivity when using blood samples for diagnosis of CanL. Moreover, we found for LAMP a specificity of 96.8%, a sensitivity of 86.2%, and a substantial agreement (*κ* = 0.80) with Rt-PCR. However, one limit of the commercial LAMP kit used in our study was the interpretation of the results by the software provided with the kit, because two samples were registered as positive (with a sign ‘plus’ near the identification of the sample) but the curve was not sigmoid. For this reason, it would be better not relying on the results recorded automatically, but it would be suggested to analyze the graph obtained also by visual inspection. 

Gao et al. [24] pointed out that one of the main limits of the LAMP methods, available at the moment, is the lack of universal primers to detect all the strains of *Leishmania infantum*. The authors reported that also, if their LAMP protocol was very sensitive (1 fg of DNA) and specific (97%), it was useful only to detect the *L. infantum* Chinese strains [8,24]. For this reason, it could be interesting to use the commercial LAMP by Avantech Group to detect strains from different areas. In addition, this new commercial LAMP kit could also be compared with the commercially available LAMP Loopamp^TM^ assay, actually used only to detect *Leishmania* in humans (Eiken Chemical Co., Tokyo, Japan).

Moreover, as also reported by other authors, it is important to point out that information provided by molecular analysis should not be separated from the data obtained from clinical signs and serological evaluations, because a positive PCR indicates a *Leishmania* infection, but not necessarily the disease development [5]. In fact, comparisons performed for the diagnosis of visceral leishmaniosis (VL) in humans between a commercial LAMP kit (EikenChemical Co., Tokyo, Japan) and other molecular and/or serological techniques, showed that LAMP could be included in the algorithm for VL, and could be used to support other laboratory findings [29,30]. 

## 5. Conclusions

An integrated approach must be used for the diagnosis of leishmaniosis, including clinical, molecular, and serological evaluations. For these reasons, LAMP could be useful, especially in veterinary clinics, for rapid screening of CanL, in the meantime, to obtain quantitative results by IFAT and/or Rt-PCR from experienced diagnostic laboratories. However, further studies should be performed to confirm these findings, increasing the number of tested dogs, involving a higher number of asymptomatic subjects. 

## Figures and Tables

**Table 1:** Frequency of clinical and clinicopathological alterations detected in chosen dogs.

Alterations	Prevalence (%)	95% CI
Lymphadenopathy	82.3	64.8–92.6
Increasing of total proteins and low A/G ratio	70.6	52.2–84.3
Skin lesions	58.8	40.8–74.9
Ocular lesions	50.0	32.8–67.3
Non-regenerative anemia	50.0	32.8–67.3
Thrombocytopenia	50.0	32.8–67.3
Renal parameters (AZO, CRE, alterations proteins/urinary creatinine)	20.6	9.3–38.4
Weight loss	17.7	7.4–35.2
Onycogryphosis	11.8	3.8–28.4
Leukocytosis/leukopenia	11.8	3.8–28.4
Increasing of transaminases	5.9	1.0–21.1
Arthritis	2.9	0.2–17.1
Epistaxis	2.9	0.2–17.1
Splenomegaly	2.9	0.2–17.1

**Table 2:** Performances of molecular techniques (LAMP and Rt-PCR) tested on lymph node samples and 95% Confidence intervals (95% CI).

Performance	Molecular Techniques on Lymph Node Samples	
	LAMP	Rt-PCR
	% (95% CI)	% (95% CI)
Specificity	96.8% (81.5–99.8)	100% (86.3–99.7)
Sensitivity	86.2% (67.4–95.5)	96.6% (80.4–99.8)
NPV	88.2% (71.6–96.2)	96.9% (82.0–99.8)
PPV	96.2% (74.4–99.8)	100% (85.0–99.7)
Time to obtain results	1 h	1 h, 15 min

**Table 3:** Comparison of results obtained by LAMP and Rt-PCR for the three sample sources examined: lymph nodes, blood, and conjunctival swabs.

	Lymph Nodes			Blood			Conjunctival Swabs		
	Rt-PCR			Rt-PCR			Rt-PCR		
**LAMP**	Positive	Negative	Total	Positive	Negative	Total	Positive	Negative	Total
Positive	24	2	26	0	0	0	10	2	12
Negative	4	30	34	3	57	60	1	47	48
Total	28	32	60	3	57	60	11	49	60

**Table 4:** Mean of *Leishmania* amastigotes/mL in the 28 Rt-PCR positive samples, divided in three range classes: low (5–100 amastigotes/mL), medium (101–1000 amastigotes/mL) and high (1001–78,000 amastigotes/mL) level of infection.

Samples (n)	*Leishmania* amastigotes/mL	
	Min–Max	Mean
11	5–100	26.8
8	101–1000	575.6
9	1001–78,000	30,022.2

**Table 5:** Performances of serological techniques tested.

Performance	Serological Techniques		
	IFAT	ELISA	SNAP
	% (95% CI)	% (95% CI)	% (95% CI)
Specificity	100% (83.4–99.6)	88.0% (67.7–96.9)	92.0% (72.5–98.6)
Sensitivity	97.2% (83.8–99.9)	94.3% (79.5–99.0)	62.9% (45.0–78.0)
NPV	96.2% (78.4–99.8)	91.7 (71.5–98.5)	63.7 (46.2–78.7)
PPV	100% (87.4–99.7)	91.7% (76.4–97.8)	91.7 (71.5–98.5)
Time to obtain results	90 min	150 min	10 min

**Table 6:** Comparison of results obtained by IFAT, ELISA, and SNAP *Leishmania* test.

	ELISA			SNAP		
IFAT	Positive	Negative	Total	Positive	Negative	Total
Positive	33	1	34	22	12	34
Negative	3	23	26	2	24	26
Total	36	24	60	24	36	60

## References

[1] Desjeux P. (2004). Leishmaniasis. Nat. Rev. Microbiol..

[2] Miró G., Petersen C., Cardoso L., Bourdeau P., Baneth G., Solano-Gallego L., Pennisi M.G., Ferrer L., Oliva G. (2017). Novel Areas for Prevention and Control of Canine Leishmaniosis. Trends Parasitol..

[3] Moreno J., Alvar J. (2002). Canine leishmaniasis: Epidemiological risk and the experimental model. Trends Parasitol..

[4] Athanasiou L.V., Kontos V.I., Saridomichelakis M.N., Rallis T.S., Diakou A. (2012). A cross-sectional sero-epidemiological study of canine leishmaniasis in Greek mainland. Acta Trop..

[5] Solano-Gallego L., Miró G., Koutinas A., Cardoso L., Pennisi M.G., Ferrer L., Bourdeau P., Oliva G., Baneth G., The LeishVet Group (2011). LeishVet guidelines for the practical management of canine leishmaniosis. Parasites Vectors.

[6] Villanueva-Saz S., Basurco A., Martín V., Fernández A., Loste A., Verde M.T. (2019). Comparison of a qualitative immunochromatographic test with two quantitative serological assays for the detection of antibodies to *Leishmania infantum* in dogs. Acta Vet. Scand..

[7] Maia C., Campino L. (2018). Biomarkers Associated with *Leishmania infantum* Exposure, Infection, and Disease in Dogs. Front. Cell. Infect. Microbiol..

[8] Chukwunonso O.N., Hirotomo K., Peters N.C. (2019). Loop-mediated isothermal amplification (LAMP): An advanced molecular point-of-care technique for the detection of *Leishmania* infection. PLoS Negl. Trop. Dis..

[9] Paltrinieri S., Gradoni L., Roura X., Zatelli A., Zini E. (2016). Laboratory tests for diagnosing and monitoring canine leishmaniasis. Vet. Clin. Pathol..

[10] Calzada J.E., Saldaña A., Gonzalez K., Rigg C., Pineda V., Santamaria A.M., Rodriguez I., Gottdenker N.L., Laurenti M.D., Chaves L.F. (2015). Cutaneous Leishmaniasis in dogs: Is high seroprevalence indicative of a reservoir role?. Parasitology.

[11] Albuquerque A., Campino L., Cardoso L., Cortes S. (2017). Evaluation of four molecular methods to detect *Leishmania* infection in dogs. Parasites Vectors.

[12] Notomi T., Okayama H., Masubuchi H., Yonekawa T., Watanabe K., Amino N., Hase T. (2000). Loop-mediated isothermal amplification of DNA. Nucleic Acids Res..

[13] Hara-Kudo Y., Konishi N., Ohtsuka K., Hiramatsu R., Tanaka H., Konuma H., Takatori K. (2008). Detection of verotoxigenic *Escherichia coli* O157 and O26 in food by plating methods and LAMP method: A collaborative study. Int. J. Food Microbiol..

[14] Wang F., Jiang L., Yang Q., Prinyawiwatkul W., Ge B. (2012). Rapid and specific detection of Escherichiacoli serogroups O26, O45, O103, O111, O121, O145, and O157 in ground beef, beef trim, and produce by loop-mediated isothermal amplification. Appl. Environ. Microbiol..

[15] Yamazaki W., Ishibashi M., Kawahara R., Inoue K. (2008). Development of a loop mediated isothermal amplification assay for sensitive and rapid detection of *Vibrio parahaemolyticus*. BMC Microbiol..

[16] Ren W., Renault T., Cai Y., Wang C. (2010). Development of a loop-mediated isothermal amplification assay for rapid and sensitive detection of ostreid herpesvirus 1 DNA. J. Virol. Methods.

[17] Ikadai H., Tanaka H., Shibahara N., Matsuu A., Uechi M., Itoh N., Oshiro S., Kudo N., Igarashi I., Oyamada T. (2004). Molecular evidence of infections with *Babesia gibsoni* parasites in Japan and evaluation of the diagnostic potential of a loop-mediated isothermal amplification method. J. Clin. Microbiol..

[18] Poon L.L., Wong B.W., Ma E.H., Chan K.H., Chow L.M., Abeyewickreme W., Tangpukdee N., Yuen K.Y., Guan Y., Looareesuwan S. (2006). Sensitive and inexpensive molecular test for falciparum malaria: Detecting *Plasmodium falciparum* DNA directly from heat-treated blood by loopmediated isothermal amplification. Clin. Chem..

[19] Karanis P., Thekisoe O., Kiouptsi K., Ongerth J., Igarashi I., Inoue N. (2007). Development and preliminary evaluation of a loop-mediated isothermal amplification procedure for sensitive detection of cryptosporidium oocysts in fecal and water samples. Appl. Environ. Microbiol..

[20] Njiru Z.K., Mikosza A.S., Armstrong T., Enyaru J.C., Ndung’u J.M., Thompson A.R. (2008). Loop-mediated isothermal amplification (LAMP) method for rapid detection of *Trypanosoma brucei rhodesiense*. PLoS Negl. Trop. Dis..

[21] Plutzer J., Karanis P. (2009). Rapid identification of *Giardia duodenalis* by loop-mediated isothermal amplification (LAMP) from faecal and environmental samples and comparative findings by PCR and real-time PCR methods. Parasitol. Res..

[22] Kumagai T., Furushima-Shimogawara R., Ohmae H., Wang T.P., Lu S., Chen R., Wen L., Ohta N. (2010). Detection of early and single infections of *Schistosoma japonicum* in the intermediate host snail, *Oncomelania hupensis*, by PCR and loop-mediated isothermal amplification (LAMP) assay. Am. J. Trop. Med. Hyg..

[23] Durand L., La Carbona S., Geffard A., Possenti A., Dubey J.P., Lalle M. (2019). Comparative evaluation of loop-mediated isothermal amplification (LAMP) vs qPCR for detection of *Toxoplasma gondii* oocysts DNA in mussels. Exp. Parasitol..

[24] Gao C.H., Ding D., Wang J.Y., Steverding D., Wang X., Yang Y.T., Shi F. (2015). Development of a LAMP assay for detection of *Leishmania infantum* infection in dogs using conjunctival swab samples. Parasites Vectors.

[25] Dixit K.K., Verma S., Singh O.P., Singh D., Singh A.P., Gupta R., Negi N.S., Das P., Sundar S., Singh R. (2018). Validation of SYBR green I based closed tube loop mediated isothermal amplification (LAMP) assay and simplified direct-blood-lysis (DBL)-LAMP assay for diagnosis of visceral leishmaniasis (VL). PLoS Negl. Trop. Dis..

[26] De Lima Celeste J.L.L., Caldeira R.L., Pires S.D.F., Silveira K.D., Soares R.P., de Andrade H.M. (2019). Development and evaluation of a loop-mediated isothermal amplification assay for rapid detection of *Leishmania amazonensis* in skin samples. Exp. Parasitol..

[27] de Avelar D.M., Carvalho D.M., Rabello A. (2019). Development and Clinical Evaluation of Loop-Mediated Isothermal Amplification (LAMP) Assay for the Diagnosis of Human Visceral Leishmaniasis in Brazil. Biomed. Res. Int..

[28] Chaouch M., Mhadhbi M., Limam S., Darghouth M.A., Benabderrazak S. (2018). Development and Evaluation of a Loop-mediated Isothermal Amplification Assay for Rapid Detection of *Theileria annulata* Targeting the Cytochrome B Gene. Iran. J. Parasitol..

[29] Mukhtar M., Ali S.S., Boshara S.A., Albertini A., Monnerat S., Bessell P., Mori Y., Kubota Y., Ndung’u J.M., Cruz I. (2018). Sensitive and less invasive confirmatory diagnosis of visceral leishmaniasis in Sudan using loop-mediated isothermal amplification (LAMP). PLoS Negl. Trop. Dis..

[30] Ibarra-Meneses A.V., Cruz I., Chicharro C., Sánchez C., Biéler S., Broger T., Moreno J., Carrillo E. (2018). Evaluation of fluorimetry and direct visualization to interpret results of a loop-mediated isothermal amplification kit to detect Leishmania DNA. Parasites Vectors.

[31] Adams E.R., Schoone G., Versteeg I., Gomez M.A., Diro E., Mori Y., Perlee D., Downing T., Saravia N., Assaye A. (2018). Development and Evaluation of a Novel Loop-Mediated Isothermal Amplification Assay for Diagnosis of Cutaneous and Visceral Leishmaniasis. J. Clin. Microbiol..

[32] Schallig H.D.F.H., Hu R.V.P., Kent A.D., van Loenen M., Menting S., Picado A., Oosterling Z., Cruz I. (2019). Evaluation of point of care tests for the diagnosis of cutaneous leishmaniasis in Suriname. BMC Infect. Dis..

[33] Von Elm E., Altman D.G., Egger M., Pocock S.J., Gøtzsche P.C., Vandenbroucke J.P. (2008). The Strengthening the Reporting of Observational Studies in Epidemiology (STROBE) Statement: Guidelines for Reporting Observational Studies. J. Clin. Epidemiol..

[34] Vitale F., Reale S., Vitale M., Petrotta E., Torina A., Caracappa S. (2004). TaqMan-based detection of *Leishmania infantum* DNA using canine samples. Ann. N. Y. Acad. Sci..

[35] Cohen J.F., Korevaar D.A., Altman D.G., Bruns D.E., Gatsonis C.A., Hooft L., Irwig L., Levine D., Reitsma J.B., de Vet H.C.W. (2016). STARD 2015 guidelines for reporting diagnostic accuracy studies: Explanation and elaboration. BMJ Open.

[36] Reitsma J.B., Rutjes A.W.S., Khan K.S., Coomarasamy A., Bossuyt P.M. (2009). A review of solutions for diagnostic accuracy studies with an imperfect or missing reference standard. J. Clin. Epidemiol..

[37] Thrusfield M. (2007). Veterinary Epidemiology.

[38] OIE (2018). Terrestrial Manual. Leishmaniosis.

[39] Farahmand M., Nahrevanian H. (2016). Application of Recombinant Proteins for Serodiagnosis of Visceral Leishmaniasis in Humans and Dogs. Iran. Biomed. J..

[40] Branscum A.J., Gardner I.A., Johnson W.O. (2005). Estimation of diagnostic-test sensitivity and specificity through Bayesian modeling. Prev. Vet. Med..

[41] Pennisi M.G., Reale S., Giudice S.L., Masucci M., Caracappa S., Vitale M., Vitale F. (2005). Real-time PCR in dogs treated for leishmaniasis with allopurinol. Vet. Res. Commun..

[42] Mori Y., Kanda H., Notomi T. (2013). Loop-mediated isothermal amplification (LAMP): Recent progress in research and development. J. Infect. Chemother..

[43] Sriworarat C., Phumee A., Mungthin M., Leelayoova S., Siriyasatien P. (2015). Development of loop-mediated isothermal amplification (LAMP) for simple detection of Leishmania infection. Parasites Vectors.

[44] Castagnaro M., Crotti A., Fondati A., Gradoni L., Lubas G., Maroli M., Oliva G., Paltrinieri S., Solano-Gallego L., Roura X. (2007). Leishmaniosi canina: Linee guida su diagnosi, stadiazione, terapia, monitoraggio e prevenzione. Parte I: Approccio diagnostico e classificazione del paziente leishmaniotico e gestione del paziente proteinurico. Veterinaria.

